# Heavy Metal Pollution from Gold Mines: Environmental Effects and Bacterial Strategies for Resistance

**DOI:** 10.3390/ijerph13111047

**Published:** 2016-10-26

**Authors:** Muibat Omotola Fashola, Veronica Mpode Ngole-Jeme, Olubukola Oluranti Babalola

**Affiliations:** 1 Food Security and Safety Niche Area, Faculty of Agriculture, Science and Technology, North-West University, Private Bag X2046, Mmabatho 2735, South Africa; 24804460@nwu.ac.za (M.O.F.); olubukola.babalola@nwu.ac.za (O.O.B.); 2 Department of Environmental Sciences, College of Agriculture and Environmental Sciences, UNISA, Florida, Private Bag X6 Florida, Roodepoort 1710, South Africa; vm.ngole@gmail.com

**Keywords:** bioremediation, environmental pollution, metal toxicity, mine wastes

## Abstract

Mining activities can lead to the generation of large quantities of heavy metal laden wastes which are released in an uncontrolled manner, causing widespread contamination of the ecosystem. Though some heavy metals classified as essential are important for normal life physiological processes, higher concentrations above stipulated levels have deleterious effects on human health and biota. Bacteria able to withstand high concentrations of these heavy metals are found in the environment as a result of various inherent biochemical, physiological, and/or genetic mechanisms. These mechanisms can serve as potential tools for bioremediation of heavy metal polluted sites. This review focuses on the effects of heavy metal wastes generated from gold mining activities on the environment and the various mechanisms used by bacteria to counteract the effect of these heavy metals in their immediate environment.

## 1. Introduction

Increased urbanization and industrialization have led to large amounts of toxic contaminants being released into the environment worldwide. Some of these contaminants occur naturally, but anthropogenic sources, especially mining activities, have contributed significantly to their increase. Although mining provides enormous social and economic benefits to nations, the long-term adverse effects on the environment and public health cannot be overlooked [1].

Mining, mineral processing and metallurgical extraction are the three principal activities of gold mining industries which produce wastes. Mineral processing also known as beneficiation aims to physically separate and concentrate the ore mineral(s) using physical, chemical and sometimes microbiological techniques. Metallurgical extraction breaks the crystallographic bonds in the ore mineral in order to recover the desired element or compound [2]. Large quantities of waste are produced during this activity. particularly in gold mines which release over 99% of extracted ore as waste to the environment [3]. 

The use of bacteria in gold extraction, known as biomining, has received considerable attention due to the potential roles played by these bacteria in the recovery of gold from gold-bearing ores. Acidophilic, chemolithotrophic iron and sulphur oxidizing bacteria such as *Acidithiobacillus* (*At.*) *ferroxidans*, *At. thioxidans*, *Leptospirillum (L.) ferriphilum* and *L. ferroxidans*, *Sulfobacillus acidophilus Sulfolobus metallicus* have been identified and utilized in gold extraction. These bacteria help in solubilizing the sulphide matrix of the gold deposits thereby making the gold more reachable to leaching by the chemical lixiviants [4,5,6]. Biomining is known to be more environmentally friendly than many physicochemical extraction processes. In addition, the wastes generated using bacteria are less biologically reactive compared to those obtained using the physicochemical methods [5].

Tailings are the major wastes produced from gold extraction and they contain high amounts of heavy metals (HM). These metals leach out in an uncontrolled manner into surrounding environments on exposure to water or through dispersal by wind. The presence of elevated concentrations of HM in the environment is a serious health issue worldwide due to their non-degradative nature which makes them persistent and thereby exert long-term effects on the ecosystem [7]. Heavy metals affect the natural population of bacteria in the soils. This leads to loss of bacterial species responsible for nutrient cycling with a consequent negative effect on ecosystem functioning [8]. To survive in metal polluted sites, some bacteria have devised various ways to withstand the potentially deleterious conditions. They are known to develop and adopt diverse detoxifying mechanisms such as biotransformation, bioaccumulation and biosorption which can be utilized in either ex-situ or in-situ bioremediation of HM polluted sites [9]. This review focuses on environmental impacts of increasing heavy metal pollution caused by gold mining activities on human health and the environment and how bacteria interact with these metals. 

## 2. Gold Processing and Extraction and the Role Played by Bacteria

Gold mining can be open-pit or deep shaft mixed with other HM such as copper (Cu), silver (Ag) and lead (Pb). Its location determines the type of mining process to be used in extraction and the amount of wastes that will be generated. In the past, small quantities of waste were generated by mining activities because higher grade ores were being exploited. There was also limited capacity to move large quantities of materials and so the waste generated was discarded within a few meters of the mine opening or pit. Open-pit mining produces eight to 10 times as much waste as underground mines because a greater amount of topsoil, overburden and barren or waste rock has to be removed. Gold mining in South Africa over the centuries has resulted in the accumulation of thousands of voluminous tailings dumps which are scattered all over the country with lots of potentially negative impact on the environments [10].

To separate the gold (Au) from the mineral bearing rock, mercury is mixed with the ores dug from the ground or from stream beds to form an amalgam. The burning of the amalgam leads to vaporization of the elemental mercury into a toxic plume leaving the gold behind. Mercury amalgamation was the initial method used for centuries to process gold and is still in use today by artisanal and small-scale gold mining (ASGM). Globally, ASGM is the second largest source of atmospheric mercury pollution after coal combustion [11]. Another method of Au extraction uses cyanide in a two-stage process; extraction and recovery. Gold is first dissolved using cyanide in the extraction stage and the dissolved gold is then recovered from the cyanide solution by cementing with zinc or adsorption onto activated carbon. The cyanide extraction processes could be heap leach or vat/tank leach depending on the quality of the ores. In ores of higher gold content, the vat/tank leaching is employed, which involves leaching of the crushed and ground ore in large enclosed tanks equipped with agitators to dissolve the gold which then adheres to pieces of the activated carbon. The activated carbon and the gold are then stripped of the solution and the barren solution together with the leached ore are discarded. The heap leach is used for low-grade ore and involves extraction of crushed oxide gold ore piled onto plastic-lined pads with leaching solvents such as acids or cyanide to dissolve the gold which is collected at the bottom of the pad [2].

The equation below explain how cyanide dissolves gold:

4Au(*s*) + 8NaCN(*aq*) + O_2_(*g*) + 2H_2_O(*l*) → 4NaAu(CN)_2_(*aq*) + 4NaOH(*aq*)

The high demand for gold and the fluctuating gold prices have necessitated the need for processing of lower grades ores, waste rock dump materials and scrap residues. Bacteria are now increasingly being used to facilitate the extraction of metals from low grades ores and concentrates (bio mining) that cannot be economically processed by conventional methods. These bacteria help in enrichment of metals in water from gold ores and mines, in a solubilization process called bioleaching. This process occurs in Nature under suitable environmental conditions that favor the growth of the bioleaching bacteria [5]. The sulphidic nature of many gold deposits hinder accessibility of lixiviants but activity of several acidophilic, chemolithotrophic iron and sulphur oxidizing bacteria has been reported to assist in the oxidation of the sulphide matrix. The bacteria include; mesophilic iron and sulphur oxidizing *Acidithiobacillus* (*At*.) *ferroxidans*, sulphur-oxidizing *At. thioxidans*, iron-oxidizing *Leptospirillum* (*L*.) *ferriphilum* and *L. ferroxidans*, moderately thermophilic bacteria such as sulphur-oxidizing *At. caldus* and sulphur and iron oxidizing *Sulfobacillus* spp. [12,13]. These bacteria obtain energy by oxidizing ferrous iron (Fe^2+^) to ferric iron (Fe^3+^) or elemental sulphur (S^0^) or other reduced sulphur compounds to sulphuric acid (H_2_SO_4_). The released Fe^3+^ and hydrogen ions then break down the sulphide matrix [14]. This is summarized in the equations below using pyrite as a typical example of gold bearing ores:

4Fe^2+^ + O_2_ + 4H^+^→ 4Fe^3+^ + 2H_2_O (1)

2S^0^ + 3O_2_ + 2H_2_O → 4H^+^ + 2SO_4_^2−^ (2)

FeS_2_ (Au) + 2Fe^3+^ → 3Fe^2+^ + 2S^0^ + (Au) (3)

FeS_2_ (Au) + 14Fe^3+^ + 8H_2_O → 15Fe^2+^ + 2SO_4_^2−^ + 16H^+^ + Au (4)

Bio-oxidation of sulphide contained in refractory gold ores enhances liberation of gold particles from the sulphide matrix thereby rendering the gold amenable to dissolution using lixiviants such as cyanide. Bio-oxidation is a pretreatment method of gold processing that helps to decrease the use of lixiviant for gold solubilization in subsequent parts of the operation and in the long run increasing the gold yields [6]. This method is usually used in conjunction with other methods since it does not actually solubilize gold. Bacteria also excrete ligands that are capable of stabilizing gold by forming gold-rich complexes and/or colloids. Biologically produced amino acids, cyanide and thiosulphate can also aid gold solubilization [6]. Gold solubility can also be reduced with the use of bacteria that help in consuming the ligands that bind the gold or by bio-sorption, enzymatic reduction and precipitation and by using gold as micronutrient [6]. In addition, bacteria can also indirectly influence gold solubilization by enhancing the permeability of gold-bearing ores bodies [15]. 

Distinct advantages have been reported for bio-leaching of gold over traditional physicochemical methods. Microbial extraction procedures are more environmentally friendly: (1) they do not produce environmentally noxious gaseous emissions; (2) they do not require high energy consumption used during roasting or smelting; (3) they enhance extraction of low grade gold ores that are too expensive to process using conventional methods; and (4) tailings produced from bio- mining processes are less chemically and biologically active since they are already bio-leached [6].

### Characteristics of Gold Mine Tailings

Tailings are a mixture of finely ground rock that is left after retrieval of the precious mineral and water used in processing. Considerable volumes of open-dump tailings are found in many countries where environmental regulations are not strongly adhered to [16]. The chemical and physical nature of tailings particles can be likened to typical river sand and silt and their properties are determined by the nature of the ore, geochemistry, the processing method used in extracting the ore, the particle size of the crushed material and the type of chemical process used in extracting the ore [17,18]. Gold mine tailings are characterized by poor physical properties like poor aggregation, high hydraulic conductivity, fine texture and very limited cohesion ability. These properties make tailings different from soil [19,20] and the lack of cohesion is responsible for the varied moisture content and temperature seen in this toxic waste. Chemically, tailings contain up to 6% pyrite, high salinity, are nutritionally deficient with low contents of organic matter [21]. The high sulphides content result in high acidity and high metal concentrations in ground water in the vicinity of the tailings [19]. Rafiei et al. [22], reported a pH value of 7.35 in gold mine tailings in Iran, whereas Mitileni et al. [23], reported pH values of 3.25–6.28 in South Africa and Harish and David [24] pH value of 3.48–8.12 in India. Highly acidic pH has also been observed in acid mine drainage arising from gold mining activity in other studies [25,26]. The characteristic features of gold mine tailings are the elevated concentrations of toxic HM such as arsenic (As), cadmium (Cd), nickel (Ni), lead (Pb), copper (Cu), zinc (Zn), cobalt (Co) and mercury (Hg) [27]. The largest fraction of the total HM may exist as silicates [28] which are limitedly accessible to microbial life. These characteristics of gold mines result in complex stresses for the bacteria inhabiting these environments and leads to selection of different resistant bacterial species. The differences in prevailing environmental conditions, levels of contamination, geographic and geologic origin as well as the site of origin are factors determining the bacterial diversity [29].

Aside from the acidophilic mesophilic species known to be involved in bio oxidation of gold, diverse metallophilic Gram positive and negative bacteria belonging to the phylum *Proteobacteria* such as *Pseudomonas*, *Aeromonas*, *Shewanella*, *Brevundimonas*, *Agrobacterium* and *Acinetobacter* and the phylum *Firmicutes* (*Bacillus*, *Serratia*, and *Exiguobacterium*) and so on have been reported in gold mine tailings using culture-dependent techniques [30,31,32,33]. A number of studies also investigated bacterial diversity in gold mines using culture independent techniques based on bacterial 16SrRNA gene identification. Santini et al. [34] in the Northern Territory of Australia also discovered the *Agrobacterium/Rhizobium* branch of the *Proteobacteria* while Rastogi et al. [35], using the same method obtained bacteria diversity mainly composed of phylotypes related to the phylum *Proteobacteria* and other phyla *Acidobacteria*, *Actinobacteria*, *Bacteroidetes*, *Chloroflexi*, *Chlorobi*, *Firmicutes*, *Nitrospirae*, *Verrumicrobia* in deep subsurface homestake gold mine soil in the USA. 

## 3. Environmental Pollution from Gold Mine Tailings

Environmental pollution from gold mines is associated mainly with the release of harmful elements from the tailings and other mine wastes. The infiltration of water through sulphide- containing tailings piles and ponds, surface and underground workings, waste and development rock leads to leaching of large volumes of metals like Zn^2+^, Ni^2+^, Pb^2+^, AS^2+^, Cu^2+^ and sulphate ions into stream and river ecosystems [36,37]. This results in acid mine drainage (AMD) with severe detrimental effect on the receiving water bodies. Heavy metal pollution and acid mine drainage is a very important environmental concern where waste materials containing metal-rich sulfides from mining activity have been stored or abandoned [38]. Tailings and rock dumps are associated with the surface impacts which greatly affect surface and ground water quality. The underground impacts are caused by the influx of water into the underground workings and the subsequent dewatering of the aquifer [39]. Another source of environmental pollution from gold mines is the chemicals used in processing the gold. An estimated 1400 metric tons of mercury was used in 2011 by ASGM and an annual average of 1000 metric tons of inorganic Hg was discharged. One-third of this estimated value goes into the air and the rest is mixed up in heaps of tailings, soil and waterways [40]. Mercury can also be released into the environment as a result of present reprocessing of some old gold tailings dumps. Pacyna et al. [41] reported that Hg emissions in South Africa are second only to China. The cyanidation method of extraction also gives rise to the emission of hydrogen cyanide, global warming and production of huge amounts of tailings a potential source of HM due to the extraction of low-grade ores [42].

### Heavy Metal Toxicity in Gold Mine Environment

Heavy metals play a vital role in metabolic and physiological processes of plants, humans and microorganisms. Heavy metals like Zn, Cu, Ni, Co and Cr, function as micronutrients and are essential in redox-processes. They are important in the stabilization of molecules through electrostatic interactions, regulation of osmotic pressure and cofactors for numerous enzymes and electron transport chains. Hence, HM ions play an essential role in complex biochemical reactions [43]. The non-essential HM like Ag, As, Cd, Pb and Hg are of no biological importance to living organisms and are very toxic when found in the ecosystem.

The disruption and acceleration of the natural process of the geochemical cycle through anthropogenic activities like gold mining has led to most soils of rural and urban settings accumulating HM above the recommended levels [44]. Studies of the effect of HM in soil, plants and water have been reported by [38,45,46,47]. 

Elevated levels of HM in gold mine tailings greatly affects the diversity, population size, and overall activity of bacteria. Heavy metals affect the metabolism, growth and morphology of soil bacteria as a result of functional disturbance, destruction of cell membrane integrity or protein denaturation [48,49]. Bacteria are essential in the decomposition of soil organic matter and any decline in bacterial diversity or biomass may have a profound effect in nutrient absorption from the soil to plants [47]. Many studies using culture dependent and independent techniques have shown that HM contamination gives rise to shifts in microbial populations [50,51,52].

Diverse toxicological and biological effects of HMs in the environment occur as a result of the different forms (oxidation) in which the HMs exists which also relates to compounds with great variation in toxicity. The oxidation state is a function of the type and quantity of the metal’s redox potential, pH and microbial activity [53]. Heavy metal toxicity results from modification in the conformational structure of nucleic acids, proteins or by interference with oxidative phosphorylation and osmotic balance [54]. Some HM like Cd^2+^, Ag^2+^, Hg^2+^ can attach to the sulfhydrl (SH) groups of important enzymes used in microbial metabolism, thereby hindering the activity of sensitive enzymes [55]. These HMs may enter the food chain as a result of their uptake by edible plants [56].

#### Cadmium

Cadmium is one of the most toxic HM to most organisms. Its concentration in unpolluted soil is usually 1 mg/kg [57], but in gold mine tailings, concentrations ranging between 6.4 and 11.7 mg/kg have been reported in Tanzania [45]. It occurs in gold bearing orebodies as an isometric trace element in sphalerite and its concentration depends on the concentration of the sphalerite in the ore body. Cadmium is of serious concern as a result of its accumulation in the food chain, drinking water and soil. It has an exceptionally long biological half-life (>20 years), highly mobile in soil-plant systems and can also exert a great effect on the proper functioning of the ecosystems [58]. The bioavalability of Cd and associated toxicity to soil bacteria depends on the bacterial species, concentration, environmental factors, time, speciation, soil properties and ageing [59]. Cadmium affects many metabolic activities of soil bacteria such as nitrogen mineralization, carbon mineralization, CO_2_ production and enzyme activities. Negative effect of Cd at concentrations of 50 and 500 mg Cd/kg were observed on dehydrogenase activities in soil bacteria by Landi et al. [60], while Smolders et al. [61] also noted 14% decrease in nitrification activity of soil bacteria in a soil having pH 6.6 at Cd concentration of 2 mg Cd/Kg. 

#### Zinc

Zinc also occurs in gold ore bodies in the form of sphalerite (ZnS) which is often associated with galena. The average natural level of Zn in the Earth’s crust is 70 mg/kg (dry weight), ranging between 10 and 300 mg/kg [62]. In gold mine tailings, concentration ranging between 8.9 and 65.7 mg/kg have been reported in South Africa by Mitileni et al. [23] while a higher concentration of 177.56 mg/kg was reported by Bempah et al. [63] in Ghana. Though a micronutrient needed by plants, bacteria and human beings for vital cell functions, its presence beyond the normal physiological value is toxic due to its interaction with sulfhydryl groups or replacement of other essential metals in a wide range of proteins [64]. Zinc speciation is very important in determining its toxicity to bacteria because it varies considerably with pH. High concentrations of Zn show varied inhibitory or toxic effect on cellular activities and growth of bacterial cells. Mertens et al. [65], noted that the nitrification process by *Nitrosospira* sp. was reduced by 20% in soil contaminated with zinc at pH 4.8–7.5. 

#### Lead

Lead is toxic at the lowest concentration and naturally non-degradable unless it is removed from the medium where it is found. Standard mean concentration for Pb in surface soils worldwide averages 32 mg/kg with a range of 10–67 mg/kg [66] but concentration ranging between 80 mg/kg [67] and 510 mg/kg [68] have been reported in gold mine tailings. It occurs in the form of galena (PbS) in gold ore and this form is found when sulphide concentration of the ore is high [69]. Lead exists in various oxidation states (0, I, II, IV) and the most stable forms are Pb(II) and lead-hydroxy complexes. The ionic form, Pb(II) is the most reactive and most common form which forms mononuclear and polynuclear oxides and hydroxides. This ionic form together with lead oxides and hydroxides are the forms that are released into surface water, ground water and soil. Lead gains access to bacterial cells through the uptake pathways for essential divalent metals like Mn^2+^ and Zn^2+^ and exerts its toxic effects on bacterial species by changing the conformation of nucleic acids, proteins, inhibition of enzyme activity, disruption of membrane functions and oxidative phosphorylation as well as alterations of the osmotic balance of the bacterial cells [43].

#### Chromium

Chromium is widely distributed in soils and rocks where it occurs in minerals such as chromite [(Fe, Mg, Al) Cr_2_O_4_]. Chromium concentration ranges between 2 and 60 mg/kg in unpolluted soil [70] but a higher concentration of 486 mg/kg was reported in gold mine tailings in Oman [67]. Chromium is mainly found in chromate FeCr_2_O_4_ having 70% of pure Cr_2_O_3_. It can be found in the environment in several forms (with oxidation states from −2 to +6) depending on pH and redox conditions but Cr(III) and VI are the most stable forms with differing chemical and physical features as well as biological effects [71]. Chromium(III) species are less soluble and relatively immobile as a result of their adsorption to clays and oxide minerals below pH 5 while low solubility above pH 5 is as a result of Cr(OH)_3_ (S). Hexavalent chromium (Cr(VI)) is the most oxidized form, a potentially dangerous substance due to its high solubility and mobility which allows it to infiltrate biological membranes and pollute soil and water [72]. This is the form usually found at contaminated sites, its major species are chromate CrO_4_^2−^ and dichromate (Cr_2_O_7_^2−^). Studies have shown that Cr(VI) is 100 times more toxic and 1000 times more mutagenic and carcinogenic compared to Cr(III) [73]. It damages bacterial DNA and this genotoxic ability has been attributed to its intracellular reduction to Cr(III) through reactive intermediates. The two types of resulting DNA damage produced are (1) oxidative DNA damage; and (2) Cr(III)-DNA interactions [74].

#### Nickel

Nickel levels in soils greatly depend on the concentration of the parent rocks and this concentration has been estimated to range from 3 to 100 mg/kg for world soils [75]. In gold mine tailings, a higher concentration of 583 mg/kg was found by Matshusa et al. [76] in South Africa. Bitala et al. [45] also reported concentrations as high as 11,200 mg/kg in Tanzania. Nickel exists in gold bearing ore as pyrrhotite (Fe_(1−X)_S), which can contain up to 5% Ni and pentlandite (FeNi)S_8_. Other mineral sources are chalcopyrite (CuFeSz) and gersdorffite (NiAsS). It exists in the 0 and +2 oxidation states and less often in the −1, +1, +3 and +4 oxidation states. Among its species, the +4 oxidation state is known to be more toxic and carcinogenic compared to +2 [77]. Nickel toxicity arises due to its tendency to substitute other metal ions in proteins, enzymes or attach to cellular compounds [78]. It also intermingles with not less than 13 essential elements in living organisms. The major toxicity of Ni to bacterial cells include: (1) replacement of essential metal of metalloproteins; (2) attachment to catalytic residues of non-metalloenzymes; (3) allosteric inhibition of enzyme; (4) oxidative stress that enchanced DNA damage, protein impairment, lipid peroxidation along with increased titers of oxidative stress defense systems [79].

#### Arsenic

Arsenic is one of the most dangerous heavy metals of worldwide environmental concern [80] due to its potential toxicity. It occurs as arsenopyrite [FeSAs], realgar [As_2_S_2_] and orpiment [As_2_S_3_] in gold bearing rock [81]. Elevated levels of As have been reported in gold mine tailings at Obuasi, Ghana. Ahmad and Carboo [82], reported 8305 mg/kg while Bempah et al. [63] found a concentration of 1752 mg/kg. The Obuasi region has been reported to be one of the regions in the world with elevated levels of As which has been attributed to the richness of arsenopyrite (FeAsS) mineralization in the gold-bearing ore [82,83]. The highest toxicity level of As is seen in the inorganic forms As(III) and arsenate As(V) which are the predominant forms in mine tailings. The arsenate acts like phosphate and can therefore gain access to microbial cells via the transport system meant for the uptake of this essential salt. Once inside the cell, it inhibits oxidative phosphorylation due to its interference with the phosphate based energy generating processes. Arsenite, on the other hand, enters through a different path (aqua-glycerolporins) and targets a wider range of cellular processes, binding to the thiol groups in essential cellular proteins such as pyruvate dehydrogenase and 2-oxoglutarate dehydrogenase [84].

#### Copper

Copper is widely distributed in sulphides, arsenites, chlorides and carbonates in gold ores. The mean concentration of 5 to 70 mg/kg exists in unpolluted soil while higher concentrations are found in contaminated environments like mining sites. Bempah et al. [63], found a concentration of 92.17 mg/kg in gold mine tailings in Ghana. Gold mining has greatly increased Cu concentration in the environment which upon release binds to particles of organic matter, clay minerals and sesquioxides leading to great accumulation in the soil [85]. Copper exists in two states, oxidized state Cu(II), and reduced state, Cu(I). The ability to exist in these two states makes this metal potentially toxic because the conversion between Cu(II) to Cu(I) could lead to a generation of superoxide and hydroxyl radicals [86]. Excessive Cu concentration has deleterious effects on soil microbes [87]. Copper toxicity is as a result of its harmful effects on the bacterial cell membranes and nucleic acid structure as well as its ability to alter enzyme specificity and disrupt cellular functions [43].

#### Mercury

Large amounts of Hg are released into the environment as a result of its usage in gold extraction. About 1.32 kg of Hg is lost for every 1 kg of gold produced which goes directly into water, soil and streams as inorganic Hg and later converted into organic forms [76]. Several researchers have reported on its high concentration in gold mine tailings. Rafiei et al. [22] reported 100 mg/kg concentrations of Hg in Iran whereas Mathusa et al. [76] reported concentrations as high as 1920 mg/kg in Kenya. Some of the inorganic Hg that reaches aquatic ecosystems is converted by microbes into organic methylmercury (MeHg), which accumulates in fish. Mercury is also inhaled during the mining and roasting processes and dangerous levels remain suspended in air due to its volatile nature. When inhaled by humans, this could lead to a series of health conditions outlined in Table 1. Mercury compounds cause oxidative stress to bacterial cells due to imbalance between pro-oxidant and anti-oxidant homeostasis. They have high affinity for thiol group containing enzymes and proteins that serve as a line of cellular defense against Hg compounds. On gaining access to the cell, both Hg II (Hg^2+^) and MeHg form covalent bonds with cysteine residues of proteins and deplete cellular antioxidants [88]. The various toxicological effects of heavy metals in human and microbes are summarized in Table 1 and Table 2.

## 4. Bacterial Interaction with Heavy Metals

Bacteria are the most abundant microorganisms in the soil, with 10^6^–10^9^ viable cells cm^−3^ of soil. Due to their small size they have a high surface to volume ratio which affords them a large contact area for interaction with their immediate environment. At a higher concentration, metal ions are known to form toxic compounds in bacteria cells [104], and their increasing concentrations in microbial habitats caused by environmental and natural processes, has led to bacteria developing various mechanisms to withstand their presence. Bacteria are known to possess the ability to convert toxic HM into insoluble substances which enhance easy mobility and dissolution in dump-sites [105]. They accumulate HM from the environment as a result of the negative net charge of their cell envelope through a metabolism-independent passive or a metabolism-dependent active process that is determined by absorptivity of the cell envelope and ability to take up HM into the cytosol [106]. This metal accumulating ability can be utilized to remove, concentrate and recover HM from industrial effluents and mine tailings [107]. In mine tailings, the redox potential, physicochemical conditions, metal speciation and co-contaminants limit bacteria-metal interactions and bacterial activity. Despite this limitation, sulphate reducing bacteria such as *Syntrophobacter sulfatireducens*, *Syntrophus gentianae*, *Desulfobacca acetoxidans*, *Desulfosporosinus* sp. and *Desulfotomaculum* sp., have been reported in both acid base-metal tailings and pH neutral gold mine tailings where they assist in natural bioremediation of mine tailings by precipitating toxic HM and increasing pH [108,109]. The level of tolerance shown by bacteria found in various gold mine tailings contaminated environments is determined by the concentration of the HM present in such environments. Several researchers have reported bacterial interactions with metals in various HM contaminated mining sites. Anderson and Cook [31], isolated 6 members of the genera *Exiguobacterium*, *Aeromonas*, *Bacillus*, *Pseudomonas*, *Escherichia*, and *Acinetobacter* resistant to arsenate from two sites contaminated with gold mine tailings in New Zealand and observed that two of the isolates, *Exiguobacterium* strain WK6 and *Pseudomonas* strain CA1 are well adapted and gained metabolic energy from the utilization of 50 mM and 30 mM of the arsenate which increased their total cell yield two fold. Similarly, Chang et al. [32], evaluated bacterial interaction with arsenic from arsenic-contaminated gold-silver mines in the Republic of Korea and discovered 15 isolates that were able to oxidize and reduce two different species of arsenic As(V) and As(III). Two of the isolates, *Pseudomonas putida* strains OS-3 and OS-18 completely oxidize 1 mM of arsenite III to V within 35–40 h of growth, while two of the four arsenate reducers obtained *P. putida* strains RS-4 and RS-5 were able to grow and efficiently utilized 66.7 mM of arsenate V. Bacterial interaction with Hg was also investigated by Ball et al. [110] in tailing ponds located in gold mining area of El Callao, Venezuela. High rates of resistance to both inorganic Hg and organomercurials were detected among the bacterial isolates. The minimal inhibitory concentrations (MIC) determined showed a broad range of resistance levels. As much as 73.58% of the isolated bacteria strains were able to grow in the presence of 0.1 mM of mercury and when grown in the presence of 0.01, 0.02, 0.04 and 0.07 mM of MeHg, the percentage resistance were 71.5%, 59.6%, 48.08% and 30.77% respectively. El Baz et al. [111], isolated 59 HM-resistant bacteria from various abandoned mining sites in Morocco that belong to *Amycolaptosis* and *Streptomyces* genera. Their results showed different levels of HM resistance, the MIC recorded in mM was 1.66 for Pb, 0.51 for Cr and 0.53 for both Zn and Cu. Bacterial interactions with metals have several impacts on the environment, they play crucial roles in the biogeochemical cycling of toxic metals as well as in cleaning or remediating metal contaminated sites [9].

### 4.1. Effects of HM on Bacteria

Microbes are usually the first biota to be affected by HM pollution [112]. Bacterial communities have been reported to be the most affected by high HM concentration as compared to fungal communities [113]. The beneficial or detrimental effect of an HM to microbial cells is a function of its concentration and the form in which it exists in the environment. The essential metals help in building the structure of an organism or assist in metabolic functions as a component of enzymes [114]. The Presence of HMs like Zn, Cu, Ni, Co and Fe at low concentrations is fundamental for numerous microbial activities, they aid in the metabolism and redox processes [114]. Exposure to high HM concentration results in selection pressure on the microbial community leading to the establishment of HM resistant microbial populations with reduced diversity when compared to unpolluted environment. The community profile is affected by reducing the number, biomass, alteration of morphological structure and loss of activity in microbially assisted soil processes such as nitrification, denitrification and decomposition of organic matter. The decrease in diversity can also result in soil erosion due to reduced soil aggregation and poor soil structure. Heavy metals also interfere with the life cycle of microbes and causes decrease in pigmentation of microbial cells [8]. Smejkalova et al. [48], studied the effect of three HM (Zn, Cd and Pb) on colony forming unit (CFU), enzymatic activities and microbial biomass carbon: oxidisable carbon content (C-biomass: Cox ratio) of a soil’s microorganisms. They discovered that all the measured parameters were significantly affected by the HM concentrations. Considerable reduction was observed on CFU which was most significant in the spore-forming and oligotrophic bacteria. Major inhibition of C-biomass was observed in these soils and the C-biomass: ox ratio decreased with increasing soil pollution.

### 4.2. Mechanisms of Bacterial Resistance to Some Selected HM

Many bacteria are able to resist and survive HM-induced stress. When the acceptable limits of HM a bacterial cell can withstand are exceeded, mechanisms of resistance are triggered in order to survive in the adverse environment [115,116]. Heavy metal tolerant bacteria have been isolated from metal laden environments with some able to survive while others are endemic to their environment and the prevailing environmental conditions may have favored their selection [117]. The ability to survive in these extreme conditions depends on acquired biochemical and structural attributes, physiological, and/or genetic adaptation such as changes of cell, morphological and environmental alterations of metal speciation [118]. Bacteria have developed several types of resistance mechanisms which aid in maintenance of intracellular homeostasis of the vital HM and normalize resistance against toxic HM which is the principle governing bioremediation processes.

#### 4.2.1. Bioaccumulation

This is an energy-dependent HM transport system that involves the retention and concentration of HM by living cells. Metals present outside bacterial cell are transported into the cytoplasm through the cell membrane and the metal is later sequestered [119] intracellularly by metal binding metallothioneins (MTs) which form complexes with the metal. Metallothioneins are small cysteine rich metal binding proteins that are induced by HM stress conditions in bacteria. They play an important role in protecting bacterial metabolic processes catalyzed by enzymes which immobilize toxic HM. Studies have shown the presence of MTs in many cyanobacterial and bacterial strains. Metallothioneins from *Synechococcus* sp. strain PCC 6301 and *Synechococcus* sp. strain PCC 7942 (SmtA) and *Pseudomonas putida* (BmtA), *Oscillatoria brevis* (BmtA), *Anabaena* PCC 7120 (SmtA), *Pseudomonas aeruginosa* (BmtA) have been described by Blindauer et al. [120]. The *smt* locus consists of two divergently transcribed genes, SmtA and SmtB which confers resistance to Zn and Cd in *Synechococcus* spp. [121]. This mechanism is subject to environmental modification, availability and toxicity of the metal, intrinsic biochemical and structural properties as well as genetic and physiological adaptation. It includes ion pumps, ion channels, carrier mediated transport, endocytosis, complex permeation, and lipid permeation. Typical examples of this active mechanism are seen in the transport of Zn, Pb, Cu, Cr and Ni [122]. Bioremediation of metals using growing bacteria cells allow both biosorption and bioaccumulation to occur simultaneously. Several authors have reported metal bioaccumulation by bacterial cells as a promising approach for clean-up of metal polluted sites [111]. Wei et al. [33], reported intracellular accumulation of four HM by bacteria strain CCNWRS33-2 isolated from root nodule of *Lespedeza cuneate* in gold mine tailings in China. This bacterium was found to have 98.9% similarity to *Agrobacterium tumefaciens* LMG 196 by 16SrRNA. The result obtained showed that 0.101 mM of Cu was accumulated after 4 h, while Cd accumulation increased from 0.225 mM at 4 h to 0.353 mM at 12 h and Pb accumulation reached 0.2 mM at 12 h. 

Despite the promising results observed from the use of growing bacterial cells for bioremediation in many studies, there are still some significant limitations to the use of this approach in treatment of HM polluted sites. Uptake of metals by bacterial cells encounters significant practical limitations such as sensitivity of the systems to extremes of pH, high salt concentration, the availability of the contaminant to the bacteria, interactions with co-ions and requirement of external metabolic energy [123,124]. Metal interaction is an important factor that needs to be considered as a results of antagonistic and synergistic interactions of metals due to their competition for the same binding sites which determines their uptake in contaminated environments like mine tailings. 

#### 4.2.2. Biosorption

This is a non-enzymatic immobilization of HM by dead or living microbial biomass. Dead biomass is better when compared to living biomass, because it is cheaper to obtain as waste, it is not affected by nutritional supply as well as HM toxicity or unfavorable operating conditions. Bio sorption denotes the totality of all passive interactions of metal ions with the cell wall, which include adsorption reactions, surface complexation reactions and ion exchange reactions with the functional groups at the cell surface [125]. In the light of reliance on metabolism, biosorption processes can be divided into metabolism dependent and metabolism independent processes. Depending upon the area where the metal removal takes place, biosorption can be categorized as extracellular accumulation/precipitation, cell surface adsorption/precipitation, and intracellular accumulation. In viable cells, biosorption is dependent on cell metabolism because it is associated with an active defense system of microorganisms, metal is transported across the cell membrane resulting in intracellular accumulation of the metal. Metabolism-independent biosorption using dead biomass occurs due to the physicochemical interaction between the metal and the functional groups (carboxyl, imidazole, sulfhydryl, amino, phosphate, sulfate, thioether, phenol, carbonyl, amide, and hydroxyl moieties) present on the cell surface of the microbial cell. This passive uptake of metal is rapid and reversible and the examples are; physical adsorption, ion exchange, and chemical sorption. Microbial cell walls comprised of polysaccharides, proteins, glucans, chitin, mannans, and phosphomannans, have abundant metal-binding groups such as carboxyl, sulphate, phosphate, and amino groups. These ligands are known to be involved in metal chelation [126,127]. In adsorption, metal ions bind non-specifically to extracellular cell surface associated polysaccharides and proteins [122]. 

Metal uptake capability by some bacteria has been reported as successful in many studies, Dorian et al. [128], evaluated bio sorption capacity of *Delftia tsuruhatensis* isolated from mine tailings in Mexico. This bacterium showed resistance to 6 mM Pb and 25 mM Zn and maximal absorption for Pb and Zn was observed to be 0.216 mM/g and 0.207 mM/g respectively. Isotherm curves generated from equilibrium batch sorption experiments and effect of process parameters have been extensively researched [125,128,129]. In addition, desorption of adsorbed metals using dilute eluents and cyclic use of regenerated biomass has also been studied [130]. However, research that takes into consideration the physicochemical conditions seen in mine tailings such as cocktail of metals, low nutrient contents of the tailings, salinity and other important factors that dictate the effectiveness of this process for efficient metals removal in mine wastes such as tailings is limited. Also, there are still limitations on most studies carried out on biosorption because information on absorbent characterizations which is an important prerequisite for repeatability of the results is still missing. Surface characterization of the bio sorbent in terms of surface area, surface morphology, functional group and particle size has now recently been included [131,132]. Also, there is a need for more research on characterizations as well as final disposal of the bio sorbent used in order to develop a reliable biosorption process

#### 4.2.3. Biotransformation

Bacteria are able to interact with HM and alter the metal structure through mechanical and biochemical mechanisms which affect the speciation and mobility of the metal [133]. Chemical transformations of HM are brought about through many processes such as oxidation, reduction, methylation, and demethylation which are sometimes by-products of normal metabolism of the bacteria [134]. Biological transformation of metals is a significant detoxification mechanism that is carried out by different bacterial species. The biological action of bacteria on HM result in changes in valency and/or conversion of HM into organometallic compounds that are volatile or less toxic [9]. In an oxidation-reduction reaction, bacteria mobilize or immobilize metal ions, metalloid and organometallic compounds, thus promoting redox processes. Heavy metal reduction by bacteria leads to HM solubility which enhances efficient mobilization of the metal. Mobilization reduces the HM to a lower oxidation state which gives rise to metallic elements (load zero) thereby reducing the metal toxicity. For example, the oxidation of arsenite As(III) to arsenate(V) and the reduction of mercury ions to metallic mercury (Hg^2+^ to Hg^0^) greatly increases the volatility of Hg and may contribute to its transport away from the microorganism’s immediate environment. In bio methylation, the transformation of HM such as Hg, As, Cd and Pb leads to their increased mobility and suitability for involvement in processes that lead to the reduction in their toxicities. It is an enzymatic mechanism that involves the transfer of methyl group (CH_3_) to metals and metalloids. The resulting methylated compounds formed differ in solubility, volatility and toxicity compared to the original metal [135]. For example, the inorganic forms of Hg are more toxic when compared to methyl and dimethyl mercury and also the inorganic forms of As are more toxic than methylated species (acids and methyl-As dimethyl-As) [136]. Numerous studies have reported the conversion of HM by bacterial cells. Govarthanan et al. [137], reported the conversion of lead nitrate Pb(NO_3_)_2_ to lead sulphide (PbS) and lead silicon oxide (PbSiO_3_) by *Bacillus* species isolated from mine tailings. In addition to this is the extracellular conversion of Pb ions to PbS by phototrophic *Rhodobacter sphaeroides* reported by Bai and Zhang [138].

### 4.3. Genetic Determinant of Metal Resistance

Genetic determinants responsible for resistance to HM are found in several bacterial strains. These resistance determinants are mediated by the chromosomal genome, plasmids or transposons and involve many operons like czcD, nccA, pco, cop, mer, ars, etc. [139]. The resistance-encoding genes seem to be plasmid mediated mainly and these findings have led to suggestions that these plasmids are most likely to be spread by horizontal transfer [140].

Zinc resistance is mediated by two efflux mechanisms which are P-type ATPase efflux and resistance nodulation cell division (RND) driven transporter system [141]. Efflux system is the most studied of all mechanisms of metal resistance in bacteria and involves an active system of transport that actively pumps back toxic ions that entered the cell via an ATPase pump or diffusion (a chemiosmotic ion or proton pump). This mechanism is mediated by plasmids and involves the P-type ATPase which catalyzes the reactions of ATP hydrolysis forming a phosphorylated intermediate [142]. Metal is transported from the cytoplasm to the periplasmic space by the energy released from ATP hydrolysis. This mechanism is one of the pathways responsible for metal resistance in Gram-negative bacteria. Xiong et al. [143], isolated a novel and multiple metalloid resistant strain, *Comamonas testosteroni* S44 having up to 10 mM resistance to zinc. Whole genome sequencing of this bacterium, revealed 9 putative Zn^2+^ transporters (4 znt operons encoding putative 4 znt operons which encode Zn^2+^ translocating P-type ATPases and 5 czc operons encoding putative RND family protein). The RND is a family of proteins that are involved in HM transport. It pumps metal from the cytoplasm directly to the extracellular space and is powered by the proton gradient across the cell wall in Gram-negative bacteria [104,141].

*Cupriavidus metallidurans* strain CH34 that was isolated from various HM laden environments is a good example of a bacterium to describe plasmid-borne determinants. This type strain possesses two large plasmids pMOL28 and pMOL30 that contain the different types of HM resistant genes. Plasmid-borne czc confers resistance to Cd, Zn, Co, ncc to Ni, Co and Cd and cnr to Co and Ni cation efflux metal resistance operons [144]. The czc locus is located on pMOL30 which is approximately 250 kb in size while the ncc and cnr were reported to be located on pMOL28 (180 kb) [145]. The *cnr*YXHCBA operon of *R. eutropha* CH34 plasmid is the most well studied of the determinants that facilitate medium levels of (up to 10 mM ) of Ni and Co resistance [144]. The mechanism of resistance mediated by cnr is inducible which as a result of an energy-dependent efflux system driven by a chemo-osmotic proton-antiporter system [146]. Another Pb resistance operon found in *Cupriavidus metallidurans* strain CH34 is the pbr, which functions in uptake, efflux and accumulation of Pb(II). These resistance loci are made of five structural resistance genes which are: (i) pbrT, which coding for Pb(II) uptake protein; (ii) pbrA, coding for a P-type Pb(II) efflux ATPase; (iii) pbrB, coding for a predicted integral membrane protein whose function is unknown; (iv) pbrC, codes for a predicted prolipoprotein signal peptidase; and (v) pbrD gene, that codes for a Pb(II)-binding protein, was identified in a region of DNA, which was essential for functional Pb sequestration [147].

The pco and cop operon comprises of four structural genes ABCD and an additional one pcoE with two regulatory trans-acting genes pcoRS and copRS [148]. The arsenic resistance system also comprises of three genes Ars ABC. Arsenite is transported by the arsenic resistance efflux using either a two-component (ArsA and ArsB) ATPase or a single polypeptide (ArsB) which functions as a chemiosmotic transporter. The *arsC*, encodes an enzyme that converts intracellular arsenate [As(V)] to arsenite [As(III)], the substrate of the efflux system [149].

The mer operon on the other hand allows bacteria to detoxify Hg^2+^ into volatile metallic mercury through enzymatic reduction. This operon varies in structure and is made up of genes that encode the functional proteins for regulation (merR) and transport (merC, merE, merF, merG, merT) of Hg^2+^ to the cytoplasm where it is reduced by merA. The merB is also found downstream of merA, a periplasmic scavenging protein (merP) and an additional one or two regulatory proteins (MerR, MerD) [150]. The genetic determinant responsible for multiple HM (As, Pb, Cd, Hg, Ni, Co and Cu) resistance patterns observed in 45 Gram positive and Gram negative bacteria isolated from the rhizosphere of *Alyssum murale* and Ni rich soil was examined by Abou-Shanab et al. [118] using polymerase chain reaction in combination with DNA sequencing. The genes responsible for this resistance (ncc, czcD, mer, and chr) were discovered to be present in these bacteria.

### 4.4. Alteration of Cell Morphology

Another mechanism that bacteria adopt to withstand HM stress is the alteration of cell morphology. This was observed in phototropic bacteria *Pseudomonas putida* and *Enterobacter* sp. on exposure to metalloid oxyanions [151] in the presence of noxious organic compounds [152]. It was also reported that high temperature brought about morphological changes in *E. coli* [153] and *Pseudomonas pseudoalcaligenes* [154]. Exposure of bacteria to unfavorable environmental conditions encountered in polluted sites such as mine tailings with toxic HM/metalloids, highly acidic or alkaline pH and the high and low temperature observed typically induced a stress response which gives rise to characteristic changes in cell shape and arrangement. These responses assist in protection of vital processes, restoration of cellular homeostasis and increase in cellular resistance against subsequent stress challenges [155]. Chakravarty et al. [156], reported the effect of four HM (Cd, Cu, Ni and Zn) on acidophilic heterotrophic *Acidocella* strain GS19h that was isolated from a copper mine. This bacterium by passes the noxious effect of the HM by reducing its surface area in relation to volume ratio. This change was due to alteration of cell morphology as a result of the penicillin binding proteins present on the bacterial cell envelope which give shape to the bacteria cell. The divalent metals structurally resemble the calcium cation and it was proposed that the metals bind in place of calcium to the binding sites as a result of their similar ligand specificities.

## 5. Future Prospects

Considering the extreme conditions that are found in gold mine tailings, future work may look at how the resistant bacteria interact with HM in this environment. To develop an efficient bioremediation approach for gold mine tailings, better understanding of bacterial interactions with metals in this environment is required.

## 6. Conclusions

Gold mining has played a tremendous role in the growth and sustenance of the economies of many countries with a huge price to pay in the form of generation and release of toxic waste products which have profound impacts on the ecosystem. Although some HM are required for normal functioning of life processes, elevated concentrations of these metals like those found in mining environments today can be toxic to bacteria that are responsible for biogeochemical cycling of nutrients which are therefore beneficial to human health. Bacterial interactions with metals in contaminated environments have important environmental and health implications, these interactions could result in clean-up of metal-contaminated sites. Most studies on bioaccumulation have focused on accumulation of individual HM ions when exposed to test organisms. Only a limited number of studies utilized growing bacterial cells with multiple mechanisms of metal sequestration and thus may hold greater metal uptake capacities. Nevertheless, such challenges can be overcome by strain selection and supply of nutrients to support the bacteria growth. The screening and selection of metal resistant strains peculiar to contaminated environments is paramount to overcome the limitation of utilizing living cell systems. Resistant cells are anticipated to bind substantial amounts of metals which will greatly enhanced bio precipitation/intracellular accumulation and development of an efficient bioremediation process. Understanding the various ways bacteria interact with these metals can elucidate on the ability of the bacteria to remove noxious ions from the environment.

## Figures and Tables

**Table 1:** Effects of heavy metals on human health.

Metals	Effects	References
As	Peripheral vascular disease, lung, skin, kidney and bladder cancer, severe disturbances of the cardiovascular and central nervous systems which may lead to death, bone marrow depression, haemolysis, hepatomegaly, melanosis, polyneuropathy and encephalopathy may also be observed.	[89]
Cd	Bronchial and pulmonary irritation, kidney stone, liver damage, various system disorders such as nervous and immune system, blood, bone and Itai itai disease.	Satarug [90]
Cr	Skin rashes, stomach and ulcers upset respiratory problems, weakened immune systems, kidney and liver damage, alteration of genetic material, lung cancer and death chromium hinder enzyme activity, DNA damage, altered gene expression and causes mutations.	[91]
Cu	Accumulation in liver, kidney, brain and cornea leading to cellular damage and Wilsons disease, upper respiratory tract and nasal mucous membrane irritation, hemolytic anaemia, epigastric pain, nausea, dizziness, headache and death may occur.	[92]
Pb	Blood related disorders such as colic, constipation and anemia, high blood pressure, decrease of hemoglobin production, kidney, joints, reproductive and cardiovascular systems disorder, long-lasting injury to the central and peripheral nervous systems, loss of IQ, low sperm count, loss of hearing.	[93,94]
Hg	Affect gene expression, kidney damage, tremor, restlessness, anxiety, depression and sleep disturbance, paresthesia and numbness in the hands and feet while high doses may lead to death. Total brain damage can occur in early exposure while late exposure results in localized damage to the cerebellum, motor cortex and the visual cortex.	[95,96]
Ni	Hypoglycemia, asthma, nausea, headache, cancer of nasal cavity and lungs.	[97]
Zn	Tachycardia, vascular shock, dyspeptic nausea, headache, cancer of nasal cavity and lungs, asthma, vomiting, diarrhea, hypoglycemia, pancreatitis and damage of hepatic parenchyma, impairment of growth and reproduction.	[97,98]

**Table 2:** Toxic effects of HM on bacteria.

Metals	Mechanisms of Action	References
Hg, Pb, Cd	Denaturation of protein	[99]
Hg, Pb, Cd and Zn	Inhibition of cell division	[99,100]
Hg, Pb, Ni, Cu and Cd	Disruption of cell membrane	[100,101]
Hg, Pb, Cd, Cu, Ni and Zn	Inhibition of enzyme activities	[100,102]
Hg, Pb, As and Cd	Damage of Nucleic acid	[99,101]
Hg, Pb, Cd	Inhibition of transcription	[103]

## References

[1] Akabzaa T.M. (2000). Boom and Dislocation: A Study of the Social and Environmental Impacts of Mining in the Wassa West District of Ghana.

[2] Lottermoser B. (2007). Mine Wastes: Characterization, Treatment and Environmental Impacts.

[3] Adler R., Rascher J. (2007). A Strategy for the Management of Acid Mine Drainage from Gold Mines in Gauteng.

[4] Acevedo F. (2000). The use of reactors in biomining processes. Electron. J. Biotechnol..

[5] Rawlings D.E. (2002). Heavy metal mining using microbes 1. Annu. Rev. Microbiol..

[6] Reith F., Rogers S.L., McPhail D., Brugger J. Potential for the Utilisation of Micro-Organisms in Gold Processing. Proceeding of the World Gold Conference.

[7] Singh R., Gautam N., Mishra A., Gupta R. (2011). Heavy metals and living systems: An overview. Indian J. Pharmacol..

[8] Piotrowska-Seget Z., Cycoń M., Kozdroj J. (2005). Metal-tolerant bacteria occurring in heavily polluted soil and mine spoil. Appl. Soil Ecol..

[9] Gadd G.M. (2010). Metals, minerals and microbes: Geomicrobiology and bioremediation. Microbiology.

[10] Dold B. (2010). Basic Concepts in Environmental Geochemistry of Sulfidic Mine-Waste Management.

[11] Telmer K., Stapper D. (2012). A Practical Guide: Reducing Mercury Use in Artisanal and Small-Scale Gold Mining.

[12] Golyshina O.V., Yakimov M.M., Lünsdorf H., Ferrer M., Nimtz M., Timmis K.N., Wray V., Tindall B.J., Golyshin P.N. (2009). *Acidiplasma aeolicum* gen. Nov., sp. Nov., a Euryarchaeon of the family *ferroplasmaceae* isolated from a hydrothermal pool, and transfer of *Ferroplasma cupricumulans* to *Acidiplasma cupricumulans* comb. Nov.. Int. J. Syst. Evol. Microbiol..

[13] Van Hille R.P., van Wyk N., Froneman T., Harrison S.T. (2013). Dynamic Evolution of the Microbial Community in Bio Leaching Tanks.

[14] Belzile N., Chen Y.-W., Cai M.-F., Li Y. (2004). A review on pyrrhotite oxidation. J. Geochem. Explor..

[15] Brehm U., Gorbushina A., Mottershead D. (2005). The role of microorganisms and biofilms in the breakdown and dissolution of quartz and glass. Palaeogeogr. Palaeoclimatol. Palaeoecol..

[16] Baker B.J., Banfield J.F. (2003). Microbial communities in acid mine drainage. FEMS Microbiol. Ecol..

[17] Franks D.M., Boger D.V., Côte C.M., Mulligan D.R. (2011). Sustainable development principles for the disposal of mining and mineral processing wastes. Resour. Policy.

[18] Davies M.P., Rice S. An alternative to conventional tailings management—“Dry stack” filtered tailings. Proceedings of the Tailings and Mine Waste’01.

[19] Vega F., Covelo E., Andrade M., Marcet P. (2004). Relationships between heavy metals content and soil properties in minesoils. Anal. Chim. Acta.

[20] Blight G., Fourie A. (2005). Catastrophe revisited-disastrous flow failures of mine and municipal solid waste. Geotech. Geol. Eng..

[21] Vega F.A., Covelo E.F., Andrade M. (2006). Competitive sorption and desorption of heavy metals in mine soils: Influence of mine soil characteristics. J. Colloid Interface Sci..

[22] Rafiei B., Bakhtiari Nejad M., Hashemi M., Khodaei A. (2010). Distribution of heavy metals around the Dashkasan Au mine. Int. J. Environ. Res..

[23] Mitileni C., Gumbo J., Muzerengi C., Dacosta F. (2011). The distribution of toxic metals in sediments: Case study of new union gold mine tailings, Limpopo, South Africa. Mine Water—Managing the Challenges.

[24] Harish E., David M. (2015). Assessment of potentially toxic cyanide from the gold and copper mine ore tailings of Karnataka, India. Int. J. Sci. Technol..

[25] Naicker K., Cukrowska E., McCarthy T. (2003). Acid mine drainage arising from gold mining activity in Johannesburg, South Africa and environs. Environ. Pollut..

[26] Tutu H., McCarthy T., Cukrowska E. (2008). The chemical characteristics of acid mine drainage with particular reference to sources, distribution and remediation: The Witwatersrand basin, South Africa as a case study. Appl. Geochem..

[27] Da Silva E.F., Zhang C., Pinto L.S.S., Patinha C., Reis P. (2004). Hazard assessment on arsenic and lead in soils of Castromil gold mining area, Portugal. Appl. Geochem..

[28] Hayes S.M., White S.A., Thompson T.L., Maier R.M., Chorover J. (2009). Changes in lead and zinc lability during weathering-induced acidification of desert mine tailings: Coupling chemical and micro-scale analyses. Appl. Geochem..

[29] Khozhina E.I., Sherriff B. (2006). Background research of the tailings area of a Ni-Cu mine for the determination of an optimal method of revegetation. For. Snow Landsc. Res..

[30] Akcil A., Karahan A., Ciftci H., Sagdic O. (2003). Biological treatment of cyanide by natural isolated bacteria (*Pseudomonas* sp.). Miner. Eng..

[31] Anderson C.R., Cook G.M. (2004). Isolation and characterization of arsenate-reducing bacteria from arsenic-contaminated sites in New Zealand. Curr. Microbiol..

[32] Chang J.-S., Kim Y.-H., Kim K.-W. (2008). The ars genotype characterization of arsenic-resistant bacteria from arsenic-contaminated gold-silver mines in the republic of Korea. Appl. Microbiol. Biotechnol..

[33] Wei G., Fan L., Zhu W., Fu Y., Yu J., Tang M. (2009). Isolation and characterization of the heavy metal resistant bacteria CCNWRSS33-2 isolated from root nodule of *Lespedeza cuneata* in gold mine tailings in China. J. Hazard. Mater..

[34] Santini J.M., Sly L.I., Schnagl R.D., Macy J.M. (2000). A new chemolithoautotrophic arsenite-oxidizing bacterium isolated from a gold mine: Phylogenetic, physiological, and preliminary biochemical studies. Appl. Environ. Microbiol..

[35] Rastogi G., Muppidi G.L., Gurram R.N., Adhikari A., Bischoff K.M., Hughes S.R., Apel W.A., Bang S.S., Dixon D.J., Sani R.K. (2009). Isolation and characterization of cellulose-degrading bacteria from the deep subsurface of the homestake gold mine, lead, South Dakota, USA. J. Ind. Microbiol. Biotechnol..

[36] Durkin T.V., Herrmann J.G. Focusing on the Problem of Mining Wastes: An Introduction to Acid Mine Drainage, EPA Seminar Publication No. EPA/625/R-95/007. http://technology.infomine.com/enviromine/publicat/amdintro.html.

[37] Edwards K.J., Bond P.L., Gihring T.M., Banfield J.F. (2000). An archaeal iron-oxidizing extreme acidophile important in acid mine drainage. Science.

[38] Concas A., Ardau C., Cristini A., Zuddas P., Cao G. (2006). Mobility of heavy metals from tailings to stream waters in a mining activity contaminated site. Chemosphere.

[39] Banister S., Van Biljon M., Pulles W. Development of appropriate procedures for water management when planning underground mine closure—A regional approach using gold mining as a case study in. Proceedings of the WISA Mine Water Division–Mine Closure Conference.

[40] Telmer K.H., Veiga M.M. (2009). World emissions of mercury from artisanal and small scale gold mining. Mercury Fate and Transport in the Global Atmosphere.

[41] Pacyna E.G., Pacyna J., Sundseth K., Munthe J., Kindbom K., Wilson S., Steenhuisen F., Maxson P. (2010). Global emission of mercury to the atmosphere from anthropogenic sources in 2005 and projections to 2020. Atmos. Environ..

[42] Bambas-Nolen L., Birn A., Cairncross E., Kisting S., Liefferink M., Mukhopadhyay B., Shroff F., van Wyk D. (2013). Case study on extractive industries prepared for the lancer commission on global governance: Report from South Africa. Lancet.

[43] Bruins M.R., Kapil S., Oehme F.W. (2000). Microbial resistance to metals in the environment. Ecotoxicol. Environ. Saf..

[44] D’amore J., Al-Abed S., Scheckel K., Ryan J. (2005). Methods for speciation of metals in soils. J. Environ. Qual..

[45] Bitala M.F., Kweyunga C., Manoko M.L. (2009). Levels of Heavy Metals and Cyanide in Soil, Sediment and Water from the Vicinity of North Mara Gold Mine in Tarime District, Tanzania.

[46] Nematshahi N., Lahouti M., Ganjeali A. (2012). Accumulation of chromium and its effect on growth of (*Allium cepa* cv. Hybrid). Eur. J. Exp. Biol..

[47] Ndeddy Aka R.J., Babalola O.O. (2016). Effect of bacterial inoculation of strains of *Pseudomonas aeruginosa, Alcaligenes feacalis* and *Bacillus subtilis* on germination, growth and heavy metal (Cd, Cr, and Ni) uptake of *Brassica juncea*. Int. J. Phytoremed..

[48] Smejkalova M., Mikanova O., Borůvka L. (2003). Effects of heavy metal concentrations on biological activity of soil micro-organisms. Plant Soil Environ..

[49] Chakravarty R., Banerjee P.C. (2008). Morphological changes in an acidophilic bacterium induced by heavy metals. Extremophiles.

[50] Bajkić S., Narančić T., Đokić L., Đorđević D., Nikodinović-Runić J., Morić I., Vasiljević B. (2013). Microbial diversity and isolation of multiple metal-tolerant bacteria from surface and underground pits within the copper mining and smelting complex bor. Arch. Biol. Sci..

[51] Xie X., Zhu W., Liu N., Liu J. (2013). Bacterial community composition in reclaimed and unreclaimed tailings of Dexing copper mine, China. Afr. J. Biotechnol..

[52] Xie Y., Fan J., Zhu W., Amombo E., Lou Y., Chen L., Fu J. (2016). Effect of heavy metals pollution on soil microbial diversity and Bermudagrass genetic variation. Front. Plant Sci..

[53] Yong R.N., Mulligan C.N. (2003). Natural Attenuation of Contaminants in Soils.

[54] Yao J., Tian L., Wang Y., Djah A., Wang F., Chen H., Su C., Zhuang R., Zhou Y., Choi M.M.F. (2008). Microcalorimetric study the toxic effect of hexavalent chromium on microbial activity of Wuhan brown sandy soil: An in vitro approach. Ecotoxicol. Environ. Saf..

[55] Turpeinen R. (2002). Interactions between Metals, Microbes and Plants: Bioremediation of Arsenic and Lead Contaminated Soils.

[56] Alirzayeva E.G., Shirvani T.S., Alverdiyeva S.M., Shukurov E.S., Öztürk L., Ali-zade V.M., Çakmak İ. (2006). Heavy metal accumulation in *Artemisia* and foliaceous lichen species from the Azerbaijan flora. For. Snow Landsc. Res..

[57] United States Environmental Protection Agency (2001). Update of Ambient Water Quality Criteria for Cadmium.

[58] United States Environmental Protection Agency (2004). List of Contaminants and Their Maximum Contaminant Levels (MCLs).

[59] Vig K., Megharaj M., Sethunathan N., Naidu R. (2003). Bioavailability and toxicity of cadmium to microorganisms and their activities in soil: A review. Adv. Environ. Res..

[60] Landi L., Renella G., Moreno J., Falchini L., Nannipieri P. (2000). Influence of cadmium on the metabolic quotient, l-: d-glutamic acid respiration ratio and enzyme activity: Microbial biomass ratio under laboratory conditions. Biol. Fertil. Soils.

[61] Smolders E., Brans K., Coppens F., Merckx R. (2001). Potential nitrification rate as a tool for screening toxicity in metal-contaminated soils. Environ. Toxicol. Chem..

[62] Malle K.-G. (1992). Zink in der umwelt. Acta Hydrochim. Hydrobiol..

[63] Bempah C.K., Ewusi A., Obiri-Yeboah S., Asabere S.B., Mensah F., Boateng J., Voigt H.-J. (2013). Distribution of arsenic and heavy metals from mine tailings dams at Obuasi municipality of Ghana. Am. J. Eng. Res..

[64] Kox L.F., Wösten M.M., Groisman E.A. (2000). A small protein that mediates the activation of a two-component system by another two-component system. EMBO J..

[65] Mertens J., Degryse F., Springael D., Smolders E. (2007). Zinc toxicity to nitrification in soil and soilless culture can be predicted with the same biotic ligand model. Environ. Sci. Technol..

[66] Kabata-Pendias A., Pendias H. (2001). Trace Elements in Soils and Plants.

[67] Abdul-Wahab S., Marikar F. (2012). The environmental impact of gold mines: Pollution by heavy metals. Open Eng..

[68] Ogola J.S., Mitullah W.V., Omulo M.A. (2002). Impact of gold mining on the environment and human health: A case study in the Migori gold belt, Kenya. Environ. Geochem. Health.

[69] Matocha C.J., Elzinga E.J., Sparks D.L. (2001). Reactivity of Pb(II) at the Mn(III, IV) (oxyhydr) oxide-water interface. Environ. Sci. Technol..

[70] Dhal B., Thatoi H., Das N., Pandey B. (2013). Chemical and microbial remediation of hexavalent chromium from contaminated soil and mining/metallurgical solid waste: A review. J. Hazard. Mater..

[71] Oliveira H. (2012). Chromium as an environmental pollutant: Insights on induced plant toxicity. J. Bot..

[72] Sultan S., Hasnain S. (2003). Pseudomonad strains exhibiting high level Cr(VI) resistance and Cr(VI) detoxification potential. Bull. Environ. Contam. Toxicol..

[73] Costa M. (2003). Potential hazards of hexavalent chromate in our drinking water. Toxicol. Appl. Pharmacol..

[74] Sobol Z., Schiestl R.H. (2012). Intracellular and extracellular factors influencing Cr(VI) and Cr(III) genotoxicity. Environ. Mol. Mutagen..

[75] Abdullahi M., Khalid R.H., Muhammad S., Munir O., Ahmet R.M. (2015). Soil contamination, remediation and plants: Prospects and challenges. Soil Remediation and Plants.

[76] Matshusa K., Ogola J.S., Maas K. Dispersion of metals at Louis Moore gold tailings dam, Limpopo province, South Africa. Proceedings of the International Mine Water Association Symposium.

[77] Higgins S.J. (1995). Nickel 1993. Coord. Chem. Rev..

[78] Cempel M., Nikel G. (2006). Nickel: A review of its sources and environmental toxicology. Pol. J. Environ. Stud..

[79] Macomber L., Hausinger R.P. (2011). Mechanisms of nickel toxicity in microorganisms. Metallomics.

[80] Choudhury B., Chowdhury S., Biswas A.K. (2011). Regulation of growth and metabolism in rice (*Oryza sativa* L.) by arsenic and its possible reversal by phosphate. J. Plant Interact..

[81] Nriagu J., Bhattacharya P., Mukherjee A., Bundschuh J., Zevenhoven R., Loeppert R. (2007). Arsenic in Soil and Groundwater: An Overview.

[82] Ahmad K., Carboo D. (2000). Speciation of As(III) and As(V) in some Ghanaian gold tailings by a simple distillation method. Water Air Soil Pollut..

[83] Bernard K.-B., Duker A.K.A.A. (2007). Assessing the Spatial Distribution of Arsenic Concentration from Goldmine for Environmental Management at Obuasi, Ghana. Master’s Thesis.

[84] Lloyd J.R., Oremland R.S. (2006). Microbial transformations of arsenic in the environment: From soda lakes to aquifers. Elements.

[85] Ranjard L., Echairi A., Nowak V., Lejon D.P., Nouaïm R., Chaussod R. (2006). Field and microcosm experiments to evaluate the effects of agricultural Cu treatment on the density and genetic structure of microbial communities in two different soils. FEMS Microbiol. Ecol..

[86] Stern B.R. (2010). Essentiality and toxicity in copper health risk assessment: Overview, update and regulatory considerations. J. Toxicol. Environ. Health A.

[87] DellAmico E., Mazzocchi M., Cavalca L., Allievi L., Vincenza A. (2008). Assessment of bacterial community structure in a long-term copper-polluted ex-vineyard soil. Microbiol. Res..

[88] Valko M., Rhodes C., Moncol J., Izakovic M., Mazur M. (2006). Free radicals, metals and antioxidants in oxidative stress-induced cancer. Chem. Biol. Interact..

[89] World Health Organization (2001). Arsenic compounds. Environmental Health Criteria.

[90] Satarug S., Garrett S.H., Sens M.A., Sens D.A. (2011). Cadmium, environmental exposure, and health outcomes. Cienc. Saude Coletiva.

[91] Bagchi D., Stohs S.J., Downs B.W., Bagchi M., Preuss H.G. (2002). Cytotoxicity and oxidative mechanisms of different forms of chromium. Toxicology.

[92] Martınez C., Motto H. (2000). Solubility of lead, zinc and copper added to mineral soils. Environ. Pollut..

[93] Lam T.V., Agovino P., Niu X., Roché L. (2007). Linkage study of cancer risk among lead-exposed workers in New Jersey. Sci. Total Environ..

[94] Shahid M., Pinelli E., Dumat C. (2012). Review of Pb availability and toxicity to plants in relation with metal speciation; role of synthetic and natural organic ligands. J. Hazard. Mater..

[95] Weiss B., Clarkson T.W., Simon W. (2002). Silent latency periods in methylmercury poisoning and in neurodegenerative disease. Environ. Health Perspect..

[96] Curtis D., Klaassen J.L.L. (2010). Casarett & Doull’s Essentials of Toxicology.

[97] Rattan R., Datta S., Chhonkar P., Suribabu K., Singh A. (2005). Long-term impact of irrigation with sewage effluents on heavy metal content in soils, crops and groundwater—A case study. Agric. Ecosyst. Environ..

[98] Salgueiro M.J., Zubillaga M., Lysionek A., Sarabia M.I., Caro R., De Paoli T., Hager A., Weill R., Boccio J. (2000). Zinc as an essential micronutrient: A review. Nutr. Res..

[99] Bánfalvi G., Banfalvi G. (2011). Heavy metals, trace elements and their cellular effects. Cellular Effects of Heavy Metals.

[100] Khan M.S., Zaidi A., Wani P.A., Oves M. (2009). Role of plant growth promoting Rhizobacteria in the remediation of metal contaminated soils. Environ. Chem. Lett..

[101] Yuan L., Zhi W., Liu Y., Karyala S., Vikesland P.J., Chen X., Zhang H. (2015). Lead toxicity to the performance, viability, and community composition of activated sludge microorganisms. Environ. Sci. Technol..

[102] Wyszkowska J., Borowik A., Kucharski M.A., Kucharski J. (2013). Effect of cadmium, copper and zinc on plants, soil microorganisms and soil enzymes. J. Elementol..

[103] Gundacker C., Gencik M., Hengstschläger M. (2010). The relevance of the individual genetic background for the toxicokinetics of two significant neurodevelopmental toxicants: Mercury and lead. Mutat. Res./Rev. Mutat..

[104] Nies D.H. (1999). Microbial heavy-metal resistance. Appl. Microbiol. Biotechnol..

[105] Gadd G.M. (2001). Accumulation and transformation of metals by microorganisms. Biotechnology Set.

[106] Hrynkiewicz K., Baum C. (2014). Application of microorganisms in bioremediation of environment from heavy metals. Environmental Deterioration and Human Health.

[107] Malekzadeh F., Farazmand A., Ghafourian H., Shahamat M., Levin M., Colwell R. (2002). Uranium accumulation by a bacterium isolated from electroplating effluent. World J. Microbiol. Biotechnol..

[108] Liu A., Garcia-Dominguez E., Rhine E., Young L. (2004). A novel arsenate respiring isolate that can utilize aromatic substrates. FEMS Microbiol. Ecol..

[109] King J.K., Kostka J.E., Frischer M.E., Saunders F.M., Jahnke R.A. (2001). A quantitative relationship that demonstrates mercury methylation rates in marine sediments are based on the community composition and activity of sulfate-reducing bacteria. Environ. Sci. Technol..

[110] Ball M.M., Carrero P., Castro D., Yarzábal L.A. (2007). Mercury resistance in bacterial strains isolated from tailing ponds in a gold mining area near El callao (Bolívar state, Venezuela). Curr. Microbiol..

[111] El Baz S., Baz M., Barakate M., Hassani L., El Gharmali A., Imziln B. (2015). Resistance to and accumulation of heavy metals by *Actinobacteria* isolated from abandoned mining areas. Sci. World J..

[112] Gutierrez-Gines M., Hernandez A., Perez-Leblic M., Pastor J., Vangronsveld J. (2014). Phytoremediation of soils co-contaminated by organic compounds and heavy metals: Bioassays with *Lupinus luteus* L. And associated endophytic bacteria. J. Environ. Manag..

[113] Rajapaksha R., Tobor-Kapłon M., Bååth E. (2004). Metal toxicity affects fungal and bacterial activities in soil differently. Appl. Environ. Microbiol..

[114] Haferburg G., Kothe E. (2007). Microbes and metals: Interactions in the environment. J. Basic Microbiol..

[115] Kim E.-H., Rensing C., McEvoy M.M. (2010). Chaperone-mediated copper handling in the periplasm. Nat. Prod. Rep..

[116] Dupont C.L., Grass G., Rensing C. (2011). Copper toxicity and the origin of bacterial resistance—New insights and applications. Metallomics.

[117] Ahemad M., Malik A. (2012). Bioaccumulation of heavy metals by zinc resistant bacteria isolated from agricultural soils irrigated with wastewater. Bacteriol. J..

[118] Abou-Shanab R., Van Berkum P., Angle J. (2007). Heavy metal resistance and genotypic analysis of metal resistance genes in gram-positive and gram-negative bacteria present in Ni-rich serpentine soil and in the rhizosphere of *Alyssum murale*. Chemosphere.

[119] Pandey A., Nigam P. (2001). Biotechnological treatment of pollutants. Chem. Ind. Digest..

[120] Blindauer C.A., Harrison M.D., Robinson A.K., Parkinson J.A., Bowness P.W., Sadler P.J., Robinson N.J. (2002). Multiple bacteria encode metallothioneins and smtA-like zinc fingers. Mol. Microbiol..

[121] Botello-Morte L., Gonzalez A., Bes M., Peleato M., Fillat M. (2013). Functional genomics of metalloregulators in *Cyanobacteria*. Genom. Cyanobact..

[122] Rani A., Goel R. (2009). Strategies for crop improvement in contaminated soils using metal-tolerant bioinoculants. Microbial Strategies for Crop Improvement.

[123] Kaduková J., Horváthová H. (2012). Biosorption of copper, zinc and nickel from multi-ion solutions. Nova Biotechnol. Chim..

[124] Zabochnicka-Świątek M., Krzywonos M. (2014). Potentials of biosorption and bioaccumulation processes for heavy metal removal. Pol. J. Environ. Stud..

[125] Sahmoune M.N., Louhab K. (2010). Kinetic analysis of trivalent chromium biosorption by dead *Streptomyces rimosus* biomass. Arab. J. Sci. Eng..

[126] Ahalya N., Ramachandra T., Kanamadi R. (2003). Biosorption of heavy metals. Res. J. Chem. Environ..

[127] Ahluwalia S.S., Goyal D. (2007). Microbial and plant derived biomass for removal of heavy metals from wastewater. Bioresour. Technol..

[128] Dorian A.B.H., Landy I.R.B., Enrique D.-P., Luis F.-L. (2012). Zinc and lead biosorption by *Delftia tsuruhatensis*: A bacterial strain resistant to metals isolated from mine tailings. J. Water Resour. Prot..

[129] Sağ Y., Kaya A., Kutsal T. (2000). Biosorption of Lead(II), Nickel(II), and Copper(II) on *Rhizopus arrhizus* from binary and ternary metal mixtures. Sep. Sci. Technol..

[130] Dixit A., Dixit S., Goswami C. (2015). Eco-friendly alternatives for the removal of heavy metal using dry biomass of weeds and study the mechanism involved. J. Bioremed. Biodegrad..

[131] Ramrakhiani L., Majumder R., Khowala S. (2011). Removal of hexavalent chromium by heat inactivated fungal biomass of *Termitomyces clypeatus*: Surface characterization and mechanism of biosorption. Chem. Eng. J..

[132] Kirova G., Velkova Z., Gochev V. (2012). Copper(II) removal by heat inactivated *Streptomyces fradiae* biomass: Surface chemistry characterization of the biosorbent. J. BioSci. Biotech..

[133] Uroz S., Calvaruso C., Turpault M.-P., Frey-Klett P. (2009). Mineral weathering by bacteria: Ecology, actors and mechanisms. Trends Microbiol..

[134] Surjit S., Barla A., Anamika S., Sutapa B. (2014). Interplay of arsenic alteration in plant soil and water: Distribution, contamination and remediation. Glob. J. Multidiscip. Stud..

[135] Gadd G.M. (2004). Microbial influence on metal mobility and application for bioremediation. Geoderma.

[136] Tabak H.H., Lens P., Hullebusch E.D.V., Dejonghe W. (2005). Developments in bioremediation of soil and sediments polluted with metals and radionuclides–1. Microbiolal processes and mechanisms affecting bioremediation of metal contamination and influencing meal toxicity. Rev. Environ. Sci. Biotechnol..

[137] Govarthanan M., Lee K.-J., Cho M., Kim J.S., Kamala-Kannan S., Oh B.-T. (2013). Significance of autochthonous *Bacillus* sp. KK1 on biomineralization of lead in mine tailings. Chemosphere.

[138] Bai H.-J., Zhang Z.-M. (2009). Microbial synthesis of semiconductor lead sulfide nanoparticles using immobilized *Rhodobacter sphaeroides*. Mater. Lett..

[139] Nies D.H. (2003). Efflux-mediated heavy metal resistance in prokaryotes. FEMS Microbiol. Rev..

[140] Rensing C., Grass G. (2003). *Escherichia coli* mechanisms of copper homeostasis in a changing environment. FEMS Microbiol. Rev..

[141] Spain A., Alm E. (2003). Implications of microbial heavy metal tolerance in the environment. Rev. Undergrad. Res..

[142] Nies D.H. (1995). The cobalt, zinc, and cadmium efflux system CzcABC from *Alcaligenes eutrophus* functions as a cation-proton antiporter in *Escherichia coli*. J. Bacteriol..

[143] Xiong J., Li D., Li H., He M., Miller S.J., Yu L., Rensing C., Wang G. (2011). Genome analysis and characterization of zinc efflux systems of a highly zinc-resistant bacterium, *Comamonas testosteroni* S44. Res. Microbiol..

[144] Mergeay M., Monchy S., Vallaeys T., Auquier V., Benotmane A., Bertin P., Taghavi S., Dunn J., van der Lelie D., Wattiez R. (2003). *Ralstonia metallidurans*, a bacterium specifically adapted to toxic metals: Towards a catalogue of metal-responsive genes. FEMS Microbiol. Rev..

[145] Monchy S., Benotmane M.A., Janssen P., Vallaeys T., Taghavi S., van der Lelie D., Mergeay M. (2007). Plasmids Pmol28 and Pmol30 of *Cupriavidus metallidurans* are specialized in the maximal viable response to heavy metals. J. Bacteriol..

[146] Taghavi S., Delanghe H., Lodewyckx C., Mergeay M., van der Lelie D. (2001). Nickel-resistance-based minitransposons: New tools for genetic manipulation of environmental bacteria. Appl. Environ. Microbiol..

[147] Borremans B., Hobman J., Provoost A., Brown N., van Der Lelie D. (2001). Cloning and functional analysis of the pbr lead resistance determinant of *Ralstonia metallidurans* CH34. J. Bacteriol..

[148] Brown N.L., Barrett S.R., Camakaris J., Lee B.T., Rouch D.A. (1995). Molecular genetics and transport analysis of the copper-resistance determinant (pco) from *Escherichia coli* plasmid pRJ1004. Mol. Microbiol..

[149] Nies D.H., Silver S. (1995). Ion efflux systems involved in bacterial metal resistances. J. Ind. Microbiol..

[150] Lin C.C., Yee N., Barkay T. (2012). Microbial transformations in the mercury cycle. Environmental Chemistry and Toxicology of Mercury.

[151] Nepple B., Flynn I., Bachofen R. (1999). Morphological changes in phototrophic bacteria induced by metalloid oxyanions. Microbiol. Res..

[152] Neumann G., Veeranagouda Y., Karegoudar T., Sahin Ö., Mäusezahl I., Kabelitz N., Kappelmeyer U., Heipieper H.J. (2005). Cells of *Pseudomonas putida* and *Enterobacter* sp. Adapt to toxic organic compounds by increasing their size. Extremophiles.

[153] Bennett A.F., Lenski R.E., Mittler J.E. (1992). Evolutionary adaptation to temperature. I. Fitness responses of *Escherichia coli* to changes in its thermal environment. Evolution.

[154] Shi B., Xia X. (2003). Morphological changes of *Pseudomonas pseudoalcaligenes* in response to temperature selection. Curr. Microbiol..

[155] Foster J., Storz G., Hengge-Aronis R. (2000). Bacterial Stress Responses.

[156] Chakravarty R., Manna S., Ghosh A.K., Banerjee P.C. (2007). Morphological changes in an *Acidocella* strain in response to heavy metal stress. Res. J. Microbiol..

